# A nationwide survey on producer and veterinarian perceptions of the painfulness of procedures and disease states in dairy and beef cattle

**DOI:** 10.3389/fpain.2023.1059224

**Published:** 2023-02-01

**Authors:** Lily N. Edwards-Callaway, Kayleigh P. Keller, Katrina Oselinsky, Elizabeth Johnstone, Catie Cramer, Noa Román-Muñiz, Lorann Stallones, Johann F. Coetzee

**Affiliations:** ^1^ Department of Animal Sciences, Colorado State University, Fort Collins, CO, United States; ^2^ Department of Statistics, Colorado State University, Fort Collins, CO, United States; ^3^ Department of Psychology, Colorado State University, Fort Collins, CO, United States; ^4^ Department of Anatomy and Physiology, Kansas State University, Manhattan, KS, United States

**Keywords:** analgesia, castration, cow, lameness, mitigation, pain, welfare

## Abstract

Failure to adequately manage pain in cattle causes suffering and is thus a welfare concern for the livestock industry. The objectives of this study were to summarize caregiver perceptions of the painfulness of various procedures and disease conditions in cattle. This survey also assessed factors that impact the perception of painfulness and determined relationships between pain perception and mitigation in producers and veterinarians in the United States beef and dairy cattle industries. An online survey was distributed *via* organization listservs and social media groups representing beef and dairy veterinarians and producers. The survey included questions about respondent demographics and pain perception and frequency of pain mitigation use for a variety of common husbandry procedures and disease conditions in cattle less than 2 months, 2–12 months, and greater than 12 months of age. Descriptive statistics were generated, and ordinal logistic regressions were used to assess the relationship between perceived pain level, frequency of pain mitigation use, and respondent demographic factors (e.g., gender, age, and role). There was a relatively low percentage of respondents that identified there was “no pain” associated with the listed procedures and conditions. Across the majority of procedures and conditions and cattle age categories, men perceived procedures to be less painful than women (*P* < 0.05). Veterinarians and producer-veterinarians perceived procedures to be more painful than producers (*P* < 0.05) for the majority of procedures and conditions. There were some differences identified between respondent age groups in pain perception but the trends were not consistent across procedures and conditions. There was a significant positive linear trend, with greater perceived pain associated with greater likelihood of providing local and systemic analgesia for all procedures and conditions across all cattle age categories (*P* ≤ 0.02). Perception of pain is complex and multifactorial, and it influences the likelihood to treat pain in cattle. This research highlighted the importance of understanding how these factors may play a role in increasing the use of pain mitigation within the beef and dairy industries.

## Introduction

Unmanaged pain in livestock as a consequence of husbandry procedures or disease states is a welfare concern and thus an important consideration for cattle industry stakeholders (1–4). Significant research has been performed to identify repeatable indicators of pain in cattle that can subsequently be used to assess the effectiveness of various pain mitigation strategies (5–8). It has been well-established that disease states, such as lameness (9, 10), and husbandry procedures, such as castration (11–13) and disbudding (14–16) cause pain in cattle, yet adoption of pain mitigation practices for these procedures and conditions is not universal and is highly variable by management practice, disease, and industry sector (17–19).

Several survey studies have explored the provision of pain mitigation for cattle of various ages, for many conditions, and in different areas of the world (19–22). In a 2010 survey of veterinarians in the United States, Coetzee et al. (23) reported that approximately 20% of respondents used some form of pain relief for surgical castration in cattle, and 92% actually performed dehorning at the same time. Similarly, Fajt et al. (24) found that approximately 30% of respondents provided some analgesic drugs when castrating calves. In a survey of veterinarians in the United Kingdom, Remnant et al. (25) reported that 67% of respondents used local anesthesia in calves undergoing castration. It is important to note that there are different regulatory requirements governing husbandry practices (26–28) and availability of analgesics for pain mitigation of livestock across countries, which are factors that could influence analgesic use.

In the United States, expectations for pain mitigation have increasingly been included in industry guidelines for cattle care. However, there are no legislative requirements and industry-driven policies are still limited and variable across procedures and conditions, likely influencing the adoption of pain mitigation practices by veterinarians and producers alike. For example, the American Association of Bovine Practitioners' (AABP) dehorning guidelines (29) recommend that “pain management be considered the standard of care during all dehorning and disbudding procedures” and although encouraged, the castration guideline language regarding pain mitigation is not as prescriptive (30), and there is no guideline document for branding. Similarly, the FARM (Farmers Assuring Responsible Management) Animal Care program Version 4.0 requires the use of pain mitigation for disbudding (31) but defers to veterinarian recommendation for pain control associated with castration and branding. Although more recent veterinarian and producer surveys have shown an increase in reported pain mitigation use for common husbandry procedures (32, 33), most studies have not demonstrated full integration of pain mitigation into best management procedures for cattle.

There is a myriad of reasons cited for why pain management is not consistently adopted into standard husbandry protocols within the beef and dairy industries. Hewson et al. (18) reported that the majority of Canadian veterinarians in a survey regarding analgesic use in cattle, pigs and horses felt there were no long-acting, cost-effective analgesics available for use in livestock and that long or unknown withdrawal periods outweighed the benefits of use. Other factors identified by veterinarians and producers as limiting pain mitigation adoption have included side effects, duration of action, cost, and availability of options (32, 34, 35). Although these are valid concerns, there are many practical approaches to providing on-farm pain management to cattle (5, 36). Some studies have shown differences between stakeholder groups on the relative importance of pain mitigation as compared with other beef and dairy cattle welfare challenges (37–39) which perhaps also influences implementation rates.

There are also human factors that have been shown to be associated with the use of pain mitigation in cattle, and the most commonly reported factors include gender, age, and experience (17, 40, 41). The perception of pain is complex and influenced by many factors thus requiring a comprehensive approach to factor evaluation. Many of the existing studies on pain perception and pain mitigation use have focused solely on common husbandry procedures such as disbudding or dehorning (21, 33, 42) and others have included extensive lists of procedures and conditions (17, 43) with slight variations in terminology across studies. Although included in some studies, information, particularly as it relates to the United States beef and dairy industries, on some common procedures and disease states, such as branding, lameness, and bovine respiratory disease (BRD) is lacking, despite their prevalence (44–46). Additionally, few studies have explored the impact of role (i.e., veterinarian vs. producer vs. both veterinarian and producer) on pain perception and subsequent likelihood to provide pain mitigation; most studies evaluate producers and veterinarians separately. Thus, the objectives of this study were to summarize perceptions of painfulness associated with various procedures and disease conditions, assess factors that may explain differences in perception of painfulness, and assess potential relationships between perceptions of painfulness and pain mitigation practices in producers and veterinarians working in the United States beef and dairy cattle industries.

## Materials and methods

The analyses reported herein represent a subset of data not previously reported from a larger survey (19, 32, 47). The survey was developed by Colorado State University (CSU) in partnership with Kansas State University (KSU) and all methods were approved by the CSU Institutional Review Board (CSU IRB #18–7937H). The study was conducted from June to August in 2018. Methods are described in brief.

### Survey population, distribution, and content

The target population for this survey consisted of veterinarians and producers working with beef and/or dairy cattle in the United States. The survey was developed in Qualtrics survey software (Qualtrics, Provo, UT) and distributed *via* several industry membership listservs and social media groups: Farm Progress (*n* = 34,681), American Association of Bovine Practitioners (*n* = 3,628), Academy of Veterinary Consultants (*n* = 901), National Milk Producers Federation Farm Evaluators (*n* = 643), Dairy Moms Facebook group (*n* = 1,797), and Dairy Girl Network Facebook group (*n* = 4,927). The survey was distributed in an initial email to the listservs with two reminder emails following approximately one week part.

The survey included 46 questions but due to the use of branching logic, individual respondents answered a variable number of questions. (e.g., if a respondent indicated they were a veterinarian, they would see different questions than those indicating they were a producer). The only forced answer question was the initial question confirming consent to participate. No individual identifying information was collected and responses were anonymous.

Questions were adapted from a 2017 survey of veterinarians in the United Kingdom (25). Respondents were asked several demographic questions related to gender, age, role in the cattle industry (veterinarian, producer, or both), location of operation, and additional information about veterinary education. The questions of interest for this paper included a subset of questions asking about the perceived painfulness associated with a variety of potentially painful procedures asked by animal age (<2 months, 2–12 months, and >12 months). The procedures and conditions presented in this paper include the following: abdominal surgery, surgical castration, band castration, hot iron dehorning, paste disbudding, hot iron branding, freeze branding, bovine respiratory disease, and lameness. For all pain perception questions the following options were provided for each question: No Pain, Mild, Moderate, Severe, Very Severe, and Worst Pain Imaginable (this category was not included in analysis). Additionally, respondents were asked to indicate how likely they would be to provide local anesthetic and a systemic pain relief drug to cattle within the three age categories for each previously listed condition. The following options to quantify the frequency of pain mitigation provision were provided: Never, Sometimes, About half the time, Most of the time, Always, and Would not perform this procedure. Some of the procedures and conditions were not included in questions asking about local anesthetic as due to the nature of the condition a local anesthetic would not be an appropriate choice due to characteristics of the procedure or condition.

### Statistical analyses

Counts and percentage of responses for each perceived pain level were aggregated by animal age and procedure. Ordinal logistic regression with a proportional odds assumption was used to assess the relationship between perceived pain level (no or mild pain combined as the reference category) and respondent demographic factors (gender [man/woman], age [21–30, 31–40, 41–50, 51–60, 61–70, 71+ years], and role [producer, veterinarian, or producer-veterinarian]). Models were fit separately for each animal age and procedure. Statistical significance at the 0.05 level was assessed using a likelihood ratio test for each categorical predictor variable. The relationship between pain perception, respondent gender, and respondent role and the frequency of local and systemic pain mitigation was then assessed using ordinal logistic regression. The outcome measure was ordered frequency of pain mitigation, excluding responses of “Would not perform this procedure”. First, a test for linear trend in the relationship between greater perceived pain and increasing frequency of pain mitigation was conducted, while adjusting for respondent gender and age. Then adjusted odds ratios were estimated for each perceived pain level, age, and gender category. The point estimates represent the multiplicative difference in odds of providing a greater frequency of pain mitigation (i.e., answering with a greater value on the ordinal scale) for the comparison category relative to the reference group. The proportional odds assumption means that the estimated odds ratios are essentially averaged across all response levels, meaning that each estimate could be approximately interpreted as the odds ratio for answering one level higher on the response. Estimated odds ratios greater than 1 indicate greater frequency of pain mitigation compared to the reference group. Statistical analyses were conducted in R, version 4.2.0 (Vienna, Austria) using the VGAM package (48).

## Results

### Respondent demographics

A total of 1,790 surveys were received; 568 surveys were removed because they were less than 80% complete and an additional 35 surveys were removed based on the respondents' roles within the cattle industry that were deemed by the authors of this paper to be too far removed from treating and caring for cattle. The final analyses included 1,187 surveys; 497 (41.9%) were producers, 569 (47.9%) were veterinarians and 121 (10.2%) were both veterinarians and producers. The majority of respondents in each category identified as male: 80.3% of producers were male, 63.4% of veterinarians were male, and 61.2% of respondents that were both veterinarians and producers were male. For further demographic description, refer to Johnstone et al. (19). Assuming there was no overlap in survey distribution, the estimated response rate was 3.8%.

### Summary of pain perception

Table 1 shows the distributions of the perceptions of painfulness of each procedure and condition by cattle age for all survey respondents. There were a sample of respondents for each procedure and condition, albeit relatively small, that indicated cattle experienced “No Pain” in response to the listed procedures and conditions. The greatest frequency of “No Pain” response was found for paste disbudding (10.4%, <2 months; 8.4%, 2–12 months) followed by freeze branding (6.1%, <2 months; 6.8% 2–12 months; 6.4% >12 months). Surgical castration, band castration, paste disbudding, and freeze branding had the greatest percentages of respondents indicating “Mild” pain; for example, in calves <2 months age, the percentages of respondents indicating “Mild” pain were 32.8% and 40.3% for surgical and band castration, respectively. For all these procedures, the frequency of respondents indicating “Mild” pain decreased as cattle age increased, i.e., fewer individuals selected “Mild” pain for cattle >12 months of age. Dehorning, hot iron branding, and abdominal surgery showed the greatest percentage of individuals identifying “Very Severe” pain; for example, the percentage of respondents indicating hot iron branding caused “Very Severe” pain ranged from 14.5% to 21.1% across cattle age categories. Additionally, a similar percentage of respondents (14.8%) selected “Very Severe” pain for cattle greater than 12 months of age undergoing band castration.

**Table 1:** Distribution of pain perceptions (e.g., No Pain, Mild, Moderate, Severe, Very Severe) across different cattle age groups (e.g., < 2 months, 2 - 12 months, > 12 months) and procedures or conditions for all survey respondents^1^.

	Pain Level					
Abdominal Surgery	No Pain	Mild	Moderate	Severe	Very Severe	No response
< 2 mo	0.6%, 7	5.7%, 65	33.3%, 381	38.5%, 441	19.2%, 220	2.7%, 31
2 to 12 mo	0.4%, 5	5.8%, 66	32.9%, 376	37.0%, 423	20.1%, 229	3.8%, 43
>12 mo	0.4%, 4	3.7%, 41	28.5%, 319	41.9%, 469	21.7%, 243	3.9%, 44
Surgical Castration						
< 2 mo	1.5%, 17	32.8%, 380	39.6%, 459	18.3%, 212	6.0%, 70	1.7%, 20
2 to 12 mo	0.7%, 8	16.1%, 185	43.9%, 506	27.4%, 316	8.8%, 101	3.1%, 36
>12 mo	0.4%, 4	5.4%, 60	25.1%, 280	38.4%, 428	26.8%, 299	3.9%, 43
Band Castration						
< 2 mo	6.8%, 79	40.3%, 470	34.8%, 406	11.6%, 135	4.0%, 47	2.4%, 28
2 to 12 mo	4.8%, 56	28.6%, 331	40.5%, 469	16.5%, 191	5.8%, 67	3.7%, 43
>12 mo	2.1%, 24	15.8%, 177	33.8%, 380	29.0%, 326	14.8%, 166	4.5%, 50
Hot Iron Dehorning						
< 2 mo	1.0%, 11	11.7%, 131	34.1%, 381	36.4%, 407	14.5%, 162	2.3%, 26
2 to 12 mo	0.3%, 3	9.3%, 104	31.7%, 354	38.6%, 431	15.8%, 176	4.3%, 48
>12 mo	0.6%, 6	8.4%, 91	25.4%, 276	37.8%, 410	21.1%, 229	6.7%, 73
Paste Disbudding						
< 2 mo	10.4%, 122	40.5%, 474	34.1%, 399	10.0%, 117	2.0%, 23	3.1%, 36
2 to 12 mo	8.4%, 98	33.9%, 396	34.9%, 408	13.7%, 160	3.3%, 39	5.8%, 68
>12 mo	-	-	-	-	-	-
Hot Iron Branding						
< 2 mo	0.7%, 8	10.7%, 119	32.2%, 357	36.6%, 406	15.9%, 176	3.8%, 42
2 to 12 mo	0.9%, 10	11.7%, 130	32.5%, 360	35.0%, 387	14.4%, 159	5.5%, 61
>12 mo	0.9%, 10	11.9%, 132	32.6%, 362	34.0%, 378	15.0%, 167	5.7%, 63
Freeze Branding						
< 2 mo	6.1%, 72	40.5%, 475	33.5%, 393	11.9%, 140	3.9%, 46	4.1%, 48
2 to 12 mo	6.8%, 80	38.8%, 455	34.0%, 398	12.1%, 142	2.6%, 30	5.7%, 67
>12 mo	6.4%, 75	37.5%, 440	35.6%, 418	11.2%, 132	3.6%, 42	5.7%, 67
Bovine Respiratory Disease						
< 2 mo	2.9%, 34	26.1%, 308	49.2%, 580	16.3%, 192	2.9%, 34	2.7%, 32
2 to 12 mo	3.4%, 40	27.6%, 325	47.6%, 561	14.7%, 173	2.4%, 28	4.3%, 51
>12 mo	3.6, %, 42	26.8%, 316	47.3%, 558	15.3%, 180	2.7%, 32	4.4%, 52
Lameness						
< 2 mo	0.9%, 10	10.6%, 125	51.7%, 608	27.0%, 318	6.2%, 73	3.6%, 42
2 to 12 mo	0.7%, 8	9.9%, 117	50.8%, 599	28.2%, 332	6.5%, 76	3.9%, 46
>12 mo	0.4%, 5	8.4%, 98	44.8%, 524	33.3%, 390	8.7%, 102	4.4%, 51

^1^ The n was different for each procedure or condition and age category and ranged from 1,007 to 1,180.

### Factors influencing the perception of painfulness

Several factors explaining differences in the perception of painfulness were identified when analyzed as single variables. The estimated odds ratios for perceived painfulness for all procedures and conditions across cattle ages by gender, role, and age of respondent are shown in Table 2. Consistently with a few exceptions, across the majority of procedures and conditions and cattle age categories, men perceived procedures to be less painful than women (*P* < 0.05); for example, in cattle <2 months, men perceived both surgical and band castration to be less painful than women did [OR (95% CI); 0.58 (0.46–0.75) and 0.60 (0.46–0.78), respectively]. For hot iron branding (all cattle ages), there was no difference in pain perception between women and men (*P* > 0.05). This relationship was also found for abdominal surgery (2–12 months), hot iron dehorning (<2 months and 2–12 months), and lameness (2–12 months and >12 months). For the majority of procedures and conditions across cattle age categories, respondent role was associated with pain perception indicating that veterinarians and producer-veterinarians perceived procedures to be more painful than producers (*P* < 0.05); for example, for cattle <2 months and 2–12 months, veterinarians [1.58 (1.06–2.35); 2.38 (1.84–3.08)] and respondents that were both veterinarians and producers [1.29 (0.86–1.92); 2.07 (1.61–2.67)], perceived paste disbudding as more painful than producers. For abdominal surgery (<2 months and >12 months), surgical castration (>12 months), and freeze branding (all cattle ages), there was no difference in pain perception between roles (*P* > 0.05). There were some differences identified between respondent age groups in pain perception but the trends were not consistent across procedures and conditions. Abdominal surgery (>12 months), paste disbudding (<2 months), and hot iron branding (2–12 months and >12 months) were the only procedures or conditions indicating a difference between age groups in pain perception (*P* < 0.05).

**Table 2:** Estimated odds ratios from ordinal logistic regression regarding the effects of gender, respondent role in the cattle industry, and respondent age on pain perception.

		Gender	Role in cattle industry		Respondent age group				
Procedure or Condition	Cattle age (months)	Male	Producer-veterinarian	Veterinarian	31–40 years	41–50 years	51–60 years	61–70 years	70+ years
Abdominal surgery	<2	0.59 (0.46–0.77)	1.25 (0.85–1.83)	1.33 (1.05–1.69)	1.16 (0.80–1.68)	1.06 (0.71–1.59)	1.34 (0.90–1.99)	1.28 (0.85–1.93)	1.43 (0.84–2.44)
	2–12	0.80 (0.62–1.03)	1.26 (0.87–1.85)	1.40 (1.10–1.78)	0.96 (0.66–1.40)	0.93 (0.62–1.40)	1.13 (0.76–1.68)	0.98 (0.65–1.47)	1.76 (1.03–3.02)
	>12	0.74 (0.56–0.96)	0.88 (0.59–1.31)	0.95 (0.74–1.21)	1.00 (0.68–1.47)	1.02 (0.68–1.55)	1.72 (1.14–2.59)	1.29 (0.85–1.95)	2.55 (1.45–4.52)
Surgical castration	<2	0.58 (0.46–0.75)	0.69 (0.47–1.01)	1.17 (0.92–1.48)	1.28 (0.89–1.85)	0.99 (0.67–1.47)	1.58 (1.07–2.35)	1.18 (0.79–1.76)	1.11 (0.65–1.90)
	2–12	0.64 (0.49–0.83)	1.01 (0.69–1.47)	1.46 (1.14–1.86)	1.24 (0.85–1.80)	1.17 (0.78–1.77)	1.48 (0.99–2.23)	1.26 (0.84–1.89)	1.44 (0.84–2.47)
	>12	0.70 (0.54–0.92)	1.29 (0.87–1.92)	1.30 (1.02–1.66)	1.47 (1.01–2.14)	1.30 (0.86–1.97)	1.75 (1.17–2.63)	1.60 (1.07–2.42)	1.99 (1.13–3.50)
Band castration	<2	0.60 (0.46–0.78)	3.16 (2.12–4.70)	3.80 (2.93–4.96)	1.25 (0.85–1.82)	0.90 (0.59–1.36)	1.47 (0.97–2.23)	1.18 (0.78–1.80)	1.28 (0.71–2.27)
	2–12	0.62 (0.47–0.81)	2.99 (2.00–4.46)	3.88 (3.01–5.03)	1.21 (0.83–1.77)	0.97 (0.64–1.47)	1.11 (0.73–1.67)	0.99 (0.65–1.49)	1.05 (0.59–1.86)
	>12	–	–	–	–	–	–	–	–
Hot iron dehorning	<2	0.73 (0.56–0.95)	1.37 (0.94–1.99)	1.96 (1.53–2.50)	1.19 (0.82–1.73)	0.86 (0.57–1.30)	1.30 (0.87–1.95)	1.17 (0.78–1.75)	1.20 (0.70–2.04)
	2–12	0.82 (0.63–1.07)	1.87 (1.26–2.76)	2.11 (1.64–2.70)	1.10 (0.75–1.60)	0.84 (0.55–1.27)	1.29 (0.86–1.95)	1.13 (0.75–1.72)	1.17 (0.67–2.02)
	>12	0.81 (0.62–1.06)	1.45 (0.97–2.18)	2.14 (1.66–2.77)	1.26 (0.86–1.84)	0.94 (0.62–1.43)	1.43 (0.94–2.15)	1.12 (0.73–1.70)	1.73 (0.97–3.10)
Paste disbudding	<2	0.54 (0.41–0.70)	1.58 (1.06–2.35)	2.38 (1.84–3.08)	1.78 (1.20–2.64)	1.31 (0.85–2.02)	1.78 (1.17–2.73)	1.66 (1.08–2.56)	2.61 (1.46–4.64)
	2–12	0.48 (0.37–0.63)	1.29 (0.86–1.92)	2.07 (1.61–2.67)	1.08 (0.74–1.58)	0.85 (0.56–1.30)	1.24 (0.82–1.87)	1.17 (0.77–1.79)	1.46 (0.83–2.57)
	>12	–	–	–	–	–	–	–	–
Hot iron branding	<2	0.88 (0.67–1.14)	0.95 (0.65–1.39)	1.51 (1.18–1.93)	1.32 (0.91–1.93)	1.07 (0.71–1.60)	1.78 (1.19–2.67)	1.40 (0.93–2.11)^*^	1.30 (0.76–2.25)
	2–12	1.00 (0.77–1.31)	1.27 (0.87–1.87)	1.66 (1.29–2.12)	1.36 (0.94–1.99)	1.10 (0.73–1.66)	2.03 (1.35–3.05)	1.57 (1.04–2.38)	1.33 (0.76–2.31)
	>12	0.91 (0.70–1.18)	0.88 (0.60–1.30)	1.50 (1.17–1.92)	1.30 (0.89–1.89)	1.26 (0.84–1.90)	2.39 (1.60–3.59)	1.70 (1.13–2.56)	2.11 (1.20–3.76)
Freeze branding	<2	0.51 (0.39–0.67)	0.78 (0.53–1.15)	1.07 (0.83–1.37)	1.31 (0.90–1.93)	1.09 (0.72–1.67)	1.37 (0.91–2.06)	1.09 (0.72–1.65)	1.08 (0.61–1.92)
	2–12	0.50 (0.38–0.65)	0.76 (0.51–1.13)	1.03 (0.80–1.32)	1.12 (0.76–1.64)	1.17 (0.77–1.80)	1.17 (0.77–1.77)	0.97 (0.64–1.48)	1.13 (0.63–2.01)
	>12	0.57 (0.43–0.74)	0.70 (0.47–1.03)	0.94 (0.73–1.20)	1.06 (0.72–1.55)	1.03 (0.68–1.57)	1.13 (0.75–1.71)	0.96 (0.63–1.45)	1.38 (0.78–2.45)
Bovine respiratory disease	<2	0.57 (0.43–0.74)	1.08 (0.73–1.60)	1.36 (1.07–1.73)	1.32 (0.90–1.93)	1.08 (0.72–1.63)	1.27 (0.84–1.91)	1.30 (0.86–1.96)	1.15 (0.65–2.01)
	2–12	0.58 (0.44–0.76)	1.57 (1.06–2.32)	1.39 (1.09–1.78)	1.14 (0.78–1.68)	0.89 (0.59–1.35)	0.85 (0.56–1.28)	1.02 (0.67–1.55)	1.23 (0.69–2.18)
	>12	0.56 (0.43–0.73)	1.46 (1.00–2.15)	1.48 (1.16–1.90)	1.01 (0.68–1.48)	0.79 (0.52–1.20)	0.82 (0.54–1.23)	0.87 (0.57–1.32)	1.25 (0.70–2.23)
Lameness	<2	0.77 (0.59–1.00)	2.26 (1.51–3.37)	2.26 (1.76–2.92)	1.15 (0.78–1.70)	1.30 (0.85–1.98)	1.33 (0.88–2.01)	1.15 (0.75–1.76)	1.82 (1.04–3.20)
	2–12	0.97 (0.74–1.27)	2.87 (1.93–4.26)	2.31 (1.79–2.99)	0.94 (0.64–1.38)	0.89 (0.59–1.35)	0.90 (0.60–1.36)	0.96 (0.63–1.47)	1.43 (0.81–2.51)
	>12	0.87 (0.67–1.13)	2.49 (1.70–3.66)	2.07 (1.61–2.68)	0.91 (0.62–1.33)	0.87 (0.58–1.31)	0.86 (0.58–1.29)	0.77 (0.51–1.17)	1.27 (0.72–2.24)

Values represent OR (95% CI). The referent for gender was female, the referent for role in the cattle industry was producer, and the referent for respondent age group was 21–30 years old. Shaded cells indicate that the OR was significantly (*P* < 0.05) different from 1 (i.e. odds for the specified group were significantly different from odds for the referent group) for the particular variable.

### Relationship between pain perception and pain mitigation

For a detailed summary of the frequencies of both local and systemic pain mitigation use for the various procedures and conditions by cattle age, refer to Johnstone et al., (19). In brief, Johnstone et al. (19) reported that analgesic use increased with cattle age; 57.6% of respondents used pain management in calves <2 months of age, while 71.6% of respondents used pain management in cattle >12 months of age. Additionally, veterinarians had significantly greater odds of using analgesia than producers across all cattle age categories. Estimated odds ratios for local anesthetic for all procedures and conditions in all age categories are shown in Table 3. There was a significant positive linear trend, with greater perceived pain associated with greater likelihood of providing local analgesia for all procedures and conditions across all cattle age categories (*P* < 0.0001); for example, as pain perception of hot iron dehorning increased, so did the odds of providing local analgesia across all age categories [e.g., for cattle >12 months: OR (CI 95%); moderate – 1.65 (1.42–1.93); severe – 5.15 (4.43–6.00); very severe – 9.61 (8.07–11.45)]. Estimated odds ratios for systemic pain relief for all procedures and conditions in all age categories are shown in Table 4. There was a significant positive linear trend, with greater perceived pain associated with greater likelihood of providing systemic analgesia for all procedures and conditions across all cattle age categories (*P* ≤ 0.02); for example, as pain perception of lameness increased, so did the odds of providing systemic analgesia across all age categories [e.g., for cattle 2–12 months: OR (CI 95%); moderate – (4.46 (3.61–5.50); severe – 12.45 (9.92–15.62); very severe – 22.90 (16.74–31.33)]. Exact *P*-values for all likelihood ratios are provided in Supplementary Material Tables S1, S2.

**Table 3:** Estimated odds ratios from an ordinal logistic regression model for the frequency of use of local anesthetic for multiple procedures and conditions across three cattle age categories (<2 months, 2–12 months, >12 months) based on perceived pain of the procedure (moderate, severe, very severe), respondent gender, and respondent role in the cattle industry.

		Pain perception of procedure		Role in cattle industry			
Procedure or Condition	Cattle age (months)	Moderate	Severe	Very Severe	Male gender	Producer-veterinarian	Veterinarian
Abdominal surgery	<2	3.01 (2.58–3.50)	6.04 (5.13–7.11)	11.59 (9.48–14.17)	0.71 (0.63–0.81)	8.12 (6.52–10.11)	4.18 (3.78–4.63)
	2–12	2.60 (2.22–3.05)	5.55 (4.67–6.60)	9.29 (7.56–11.41)	0.56 (0.50–0.66)	8.93 (6.93–11.52)	4.26 (3.82–4.74)
	>12	1.18 (0.94–1.49)	3.04 (2.41–3.84)	3.60 (2.77–4.67)	0.37 (0.30–0.45)	10.45 (7.52–14.50)	9.29 (7.92–10.89)
Surgical castration	<2	2.48 (2.25–2.71)	8.25 (7.38–9.21)	16.95 (14.35–20.03)	0.64 (0.59–0.70)	1.06 (0.93–1.22)	1.62 (1.48–1.77)
	2–12	2.52 (2.23–2.84)	6.29 (5.54–7.13)	15.66 (13.30–18.45)	0.61 (0.56–0.65)	1.63 (1.44–1.85)	2.05 (1.88–2.23)
	>12	2.09 (1.75–2.50)	4.24 (3.56–5.05)	12.78 (10.63–15.37)	0.53 (0.49–0.57)	2.58 (2.27–2.93)	3.50 (3.21–3.82)
Band castration	<2	3.31 (2.95–3.73)	6.05 (5.17–7.08)	16.60 (13.19–20.90)	0.76 (0.69–0.84)	0.32 (0.26–0.39)	0.79 (0.70–0.88)
	2–12	2.84 (2.50–3.21)	5.87 (5.07–6.79)	11.47 (9.27–14.20)	0.71 (0.64–0.78)	0.59 (0.49–0.70)	0.91 (0.81–1.01)
	>12	–	–	–	–	–	–
Hot iron dehorning	<2	1.80 (1.59–2.04)	4.16 (3.68–4.72)	12.18 (10.37–14.28)	0.54 (0.50–0.59)	3.40 (3.01–3.83)	5.34 (4.91–5.80)
	2–12	2.55 (2.18–2.97)	6.23 (5.34–7.27)	13.46 (11.25–16.10)	0.52 (0.47–0.56)	3.80 (3.34–4.33)	6.03 (5.52–6.58)
	>12	1.65 (1.42–1.93)	5.15 (4.43–6.00)	9.61 (8.07–11.45)	0.47 (0.43–0.52)	8.44 (7.26–9.81)	8.44 (7.68–9.27)
Paste disbudding	<2	1.82 (1.70–1.95)	2.30 (2.05–2.59)	6.42 (4.69–8.80)	0.54 (0.50–0.58)	3.04 (2.73–3.39)	4.96 (4.61–5.34)
	2–12	2.37 (2.19–2.57)	3.15 (2.80–3.55)	3.47 (2.75–4.37)	0.57 (0.53–0.62)	4.49 (3.97–5.07)	6.27 (5.78–6.81)
	>12	–	–	–	–	–	–

Values represent OR (95% CI). The referent for pain perception was a combined category of No Pain and Mild pain, the referent for gender was female, the referent for role in the cattle industry was producer. The OR was significantly (*P* < 0.05) different from 1 (i.e. odds for the specified group were significantly different from odds for the referent group) for pain perception for all procedures. Shaded cells indicate that the OR was significantly (*P* < 0.05) different from 1 (i.e. odds for the specified group were significantly different from odds for the referent group) for the particular variable.

**Table 4:** Estimated odds ratios from a model for the frequency of use of systemic analgesic for multiple procedures and conditions across three cattle age categories (<2 months, 2–12 months, >12 months) based on perceived pain of the procedure (moderate, severe, very severe), respondent gender, and respondent role in the cattle industry.

		Pain perception of procedure		Role in cattle industry			
Procedure or Condition	Cattle age (months)	Moderate	Severe	Very Severe	Male gender	Producer-veterinarian	Veterinarian
Abdominal surgery	<2	3.31 (2.77–3.95)	6.86 (5.72–8.22)	14.47 (11.79–17.76)	0.63 (0.57–0.69)	1.61 (1.38–1.87)	1.32 (1.20–1.46)
	2–12	5.48 (4.48–6.71)	11.76 (9.56–14.56)	22.37 (17.74–28.21)	0.62 (0.55–0.69)	2.45 (2.05–2.94)	1.42 (1.28–1.58)
	>12	1.79 (1.45–2.20)	4.06 (3.30–5.01)	7.43 (5.91–9.35)	0.84 (0.76–0.93)	1.54 (1.31–1.82)	1.06 (0.96–1.18)
Surgical castration	<2	2.31 (2.05–2.61)	9.55 (8.32–10.96)	16.94 (13.95–20.56)	0.72 (0.65–0.79)	2.32 (1.95–2.77)	3.32 (2.94–3.76)
	2–12	3.02 (2.54–3.61)	9.10 (7.59–10.91)	27.19 (21.62–34.21)	0.67 (0.61–0.74)	2.77 (2.35–3.28)	3.21 (2.84–3.62)
	>12	2.21 (1.73–2.82)	6.52 (5.13–8.28)	15.70 (12.21–20.19)	0.57 (0.52–0.63)	5.23 (4.41–6.21)	4.37 (3.88–4.92)
Band castration	<2	2.48 (2.21–2.79)	6.62 (5.69–7.70)	11.36 (8.96–14.40)	0.61 (0.55–0.68)	0.90 (0.74–1.10)	2.07 (1.82–2.35)
	2–12	2.61 (2.24–3.03)	7.95 (6.68–9.45)	13.02 (9.85–17.21)	0.62 (0.55–0.69)	0.89 (0.72–1.11)	1.80 (1.56–2.07)
	>12	–	–	–	–	–	–
Hot iron dehorning	<2	1.64 (1.40–1.92)	3.87 (3.31–4.52)	7.50 (6.30–8.94)	0.60 (0.55–0.65)	2.39 (2.08–2.76)	2.94 (2.67–3.25)
	2–12	3.83 (3.06–4.80)	7.69 (6.16–9.62)	19.41 (15.30–24.64)	0.72 (0.66–0.79)	2.61 (2.25–3.03)	3.11 (2.80–3.44)
	>12	2.16 (1.81–2.59)	4.76 (4.00–5.68)	8.39 (6.95–10.12)	0.69 (0.63–0.75)	5.38 (4.63–6.24)	4.08 (3.69–4.51)
Paste disbudding	<2	2.08 (1.92–2.26)	3.25 (2.87–3.69)	4.96 (3.80–6.46)	0.61 (0.56–0.66)	2.21 (1.94–2.52)	2.81 (2.57–3.08)
	2–12	2.01 (1.83–2.20)	3.07 (2.71–3.47)	4.14 (3.32–5.16)	0.76 (0.70–0.83)	3.23 (2.81–3.72)	3.45 (3.14–3.81)
	>12	–	–	–	–	–	–
Hot iron branding	<2	1.75 (1.33–2.32)	3.36 (2.55–4.42)	9.00 (6.67–12.13)	0.77 (0.65–0.90)	1.48 (1.18–1.87)	1.83 (1.55–2.16)
	2–12	2.90 (2.03–4.15)	6.95 (4.88–9.88)	7.47 (4.97–11.22)	0.78 (0.65–0.94)	1.35 (1.04–1.74)	1.39 (1.16–1.68)
	>12	1.48 (1.29–1.70)	1.39 (1.22–1.59)	1.38 (1.18–1.61)	0.74 (0.67–0.82)	1.32 (1.13–1.54)	0.98 (0.89–1.08)
Freeze branding	<2	4.72 (4.04–5.52)	4.49 (3.55–5.69)	25.10 (18.15–34.71)	1.05 (0.90–1.23)	1.41 (1.12–1.77)	2.27 (1.93–2.68)
	2–12	3.69 (3.07–4.44)	8.21 (6.31–10.68)	7.16 (4.51–11.35)	1.03 (0.85–1.24)	1.62 (1.26–2.09)	1.68 (1.39–2.02)
	>12	1.31 (1.19–1.44)	1.00 (0.87–1.14)	2.37 (1.80–3.11)	0.79 (0.72–0.87)	1.39 (1.20–1.62)	1.02 (0.93–1.12)
Bovine respiratory disease	<2	2.87 (2.55–3.23)	8.47 (7.15–10.03)	7.39 (5.27–10.36)	0.50 (0.45–0.56)	2.41 (2.02–2.87)	2.15 (1.93–2.40)
	2–12	3.25 (2.88–3.67)	8.71 (7.28–10.43)	16.85 (10.44–27.19)	0.52 (0.46–0.58)	2.19 (1.82–2.63)	2.03 (1.81–2.27)
	>12	1.41 1.29–1.55)	1.92 (1.68–2.20)	1.49 (1.15–1.94)	0.84 (0.77–0.93)	1.27 (1.09–1.48)	0.96 (0.87–1.05)
Lameness	<2	3.57 (2.98–4.29)	7.13 (5.85–8.69)	16.84 (12.60–22.51)	0.65 (0.58–0.72)	2.92 (2.44–3.50)	2.55 (2.28–2.85)
	2–12	4.46 (3.61–5.50)	12.45 (9.92–15.62)	22.90 (16.74–31.33)	0.56 (0.50–0.63)	2.48 (2.04–3.00)	2.37 (2.11–2.67)
	>12	1.12 (0.95–1.32)	1.26 (1.07–1.50)	1.81 (1.47–2.24)	0.81 (0.73–0.88)	1.23 (1.08–1.46)	0.95 (0.87–1.05)

Values represent OR (95% CI). The referent for pain perception was a combined category of No Pain and Mild pain, the referent for gender was female, the referent for role in the cattle industry was producer. The OR was significantly (*P* < 0.05) different from 1 (i.e. odds for the specified group were significantly different from odds for the referent group) for pain perception for all procedures. Shaded cells indicate that the OR was significantly (*P* < 0.05) different from 1 (i.e. odds for the specified group were significantly different from odds for the referent group) for the particular variable.

## Discussion

The provision of analgesia to mitigate pain associated with painful husbandry procedures and disease states and the associated welfare impacts have been widely discussed in the beef and dairy cattle industries (1, 2, 4). Pain research in cattle has focused on various areas including validating indicators of pain (49–51), evaluating methods of pain mitigation (52–55), and quantifying the frequency of pain mitigation implementation by producers and veterinarians for various procedures (19, 23, 24, 25). Although many challenges impacting the use of pain mitigation for livestock have been identified by producers and veterinarians (18, 32), there has been more limited exploration of how perceptions of pain and demographic factors, considered separately and together, influence the implementation of pain mitigation for some key procedures and conditions relevant to beef and dairy production systems in the United States. The aim of this study was to explore the relationship between perceptions of painfulness associated with various procedures and conditions in addition to demographic factors and subsequent pain mitigation practices used by producers and veterinarians working in the United States beef and dairy cattle industries.

All the procedures or disease states included in this study have been documented to cause some level of pain in cattle [e.g., abdominal surgery (56); surgical castration (11); band castration (57); hot iron dehorning (58); paste disbudding (14); hot iron branding (55); freeze branding (59); bovine respiratory disease (60); lameness (9)]. Despite the knowledge around how painful these procedures are for animals, there were respondents in this study that indicated “no pain” would be experienced by cattle for each listed procedure or condition. This subset of respondents was relatively small compared to the other pain categories but the fact that this was the perception of some is worth noting. For example, 10.4% (*n* = 122) of respondents indicated that they felt paste disbudding caused “no pain” in calves less than two months of age. Interestingly, disbudding is one of the only procedures that has a requirement for pain mitigation in the industry animal care program for dairy cattle (31). This result is also likely in part explained by the misleading notion that younger animals feel less pain. Research has established that young animals, i.e., calves, do feel pain (8, 61, 62) but the response is expressed differently both physiologically and behaviorally as compared with older animals (12, 13, 57, 63). Additionally, research in rats and humans has demonstrated that pain and stress events experienced as infants can have long-lasting physiological impacts (64). The proportions of respondents indicating “mild pain” for several of the procedures was also relatively high when considering current knowledge about the pain associated with those procedures. For example, nearly one-third of respondents (32.8%, 380) indicated that surgical castration caused “mild pain” in calves under the age of 2 months, once again potentially confounded by the misconception that young animals feel less pain. There were also respondents that provided “no response” for certain procedures and conditions. This could be attributed to several reasons including lack of familiarity with the condition, not having an opinion about how painful the procedure was, or not using that practice, but follow up questions to identify the reasons were not asked within the survey tool.

This study demonstrated that the perception of pain significantly impacted the likelihood of respondents to provide both local and systemic analgesics to cattle across all ages for all procedures and conditions; for all procedures and conditions, with higher perceived pain strongly associated with greater frequency of providing pain mitigation. There are many factors (social, demographic, environmental, etc.) that can impact someone's perception of pain and these factors often are related and interact with each other making the perception of pain a complicated topic. When exploring effects of gender alone on pain perception, men rated the majority of procedures as less painful compared to women in the current study; hot iron branding was the only procedure that did not show an impact of gender on perception of pain across all age groups. This finding aligns with previously published research reporting that veterinarians, veterinary students, and producers who identified as women perceived certain management procedures and conditions as more painful compared to men (17, 35, 40, 43, 65–67). Perhaps an individual's perception of their own pain translates into different perception of painfulness for other beings, such as cattle. Gender differences in pain perception and prevalence of chronic pain have been evaluated extensively in human medicine (68–74) with results suggesting that women are less tolerant and more sensitive to pain than men. Specifically, research suggests women are more likely to experience recurrent pain, experience pain more frequently, feel that pain is longer lasting, and experience more severe pain than men (75). Additionally, women are more prone to experience certain painful conditions as compared to men including musculoskeletal pain, rheumatoid arthritis, neuropathic pain, facial pain, and headaches (68, 69, 75). Although these human studies point to differences in pain perception, prevalence, and tolerance, the exact factors influencing gendered differences in pain are debated and research is inconclusive regarding the causal mechanisms driving this difference which are likely multifactorial and complex.

Empathy plays an important role in perceiving and reacting to pain in both humans and animals. Overall, women are more empathic to pain in other humans and in animals (76). One study found that empathy was the best predictor of how people rated pain in dogs and concluded that gender was one of the most stable factors influencing empathy, attitudes, and the perception of pain (77). In veterinary medicine, the empathy skills of a veterinarian have been shown to impact their pain ratings, with more empathetic veterinarians identifying greater pain scores (78). The authors speculate that being more empathetic does not necessarily result in more accurate estimates of pain, however, veterinarians and producers who display greater levels of empathy have been shown to use pain mitigation techniques more frequently (40, 78). Although empathy is important for the identification and treatment of animal pain, levels of both empathy and appreciation of animal sentience seem to fluctuate during different years of veterinary studies and may be attributed to numerous reasons including desensitization over time or as a coping mechanism to deal with the anticipated emotional distress associated with veterinary medicine (79–81). Alternatively, the decrease in empathy found in both veterinary and human medicine education may be a coping mechanism used by professional students to tolerate the accumulation of negative affective experiences enabling them to remain in their chosen profession (82). One study hypothesized the greater pain scores assigned by more experienced veterinary nurses might be attributed to greater professional experience and also an accumulation of individuals' experiences with pain through childbirth or surgery (83). Future research, particularly with regard to veterinary education, should examine how and why empathy changes over time and what this means for the welfare of animals in their care.

The role that the survey respondents had in the cattle industry also influenced their perception of pain. Respondents that identified as veterinarians only or both veterinarians and producers rated pain greater than producers for almost all procedures and conditions. This finding aligns with previous studies (33, 42), especially when considering that pain perception and the provision of pain mitigation for painful procedures are correlated. For example, while the proportion of producers in the Midwestern United States providing pain control medications to calves undergoing disbudding or dehorning has increased, the odds of using pain control practices increases when veterinarians are involved in protocol development for these procedures (33). Veterinarians' training to identify pain and discomfort very likely contributes to their increased pain perception. A study conducted in the UK showed marked differences in pain perception and use of nonsteroidal anti-inflammatory drugs to manage post disbudding procedures in calves between veterinarians and farmers (42). While formal training and clinical experience contributed to veterinarians' knowledge about pain associated with various procedures and pain management strategies, cattle producers cited tradition, training courses, veterinarians, and media as their sources of knowledge with respect to pain mitigation for disbudding procedures (42). Inadequate pain recognition and ingrained farm practices were identified by veterinarians in New Zealand as obstacles to the use of pain mitigation on-farm after surgical procedures in calves, i.e., castration, disbudding and extra numerary teat removal (84). Others have noted that while producers and veterinarians shared concerns about diseases and pain management, differences in beliefs, and the capacity to address problems may influence their perspectives on cattle welfare (85). Because of differences in pain perceptions and the degree of influence that veterinarians have on animal health management, an increasing body of literature highlights the need for improved communication and collaboration between veterinarians and cattle producers to minimize barriers to appropriate pain management and to improve animal welfare on farms (33, 42, 84, 85).

In the current study, there was no consistent pattern for the influence of age on pain perception, aligning with the current literature (40, 41, 86, 87); for the majority of procedures and conditions there was no effect of age on pain perception. Survey studies with dairy and beef producers have demonstrated that older producers were more sensitive to pain in cattle than younger producers (40, 41). Additionally, a study conducted at an animal welfare symposium found that among those interested in animal welfare as indicated by their attendance at the symposium, older people displayed a stronger belief in animal sentience relating to hunger and pain across a variety of animal species (88). Conversely, Kielland et al. (86) found in a study of veterinary students that age did not influence pain perception and Raekallio et al. (65) concluded that younger veterinarians provided greater pain ratings and were more likely to provide analgesics than their older colleagues. Age could be confounded by other factors such as personal experience and year of graduation from veterinary school. Several studies have reported that both veterinarians and producers with more experience perceive certain procedures and conditions as more painful (40, 87). Although not evaluated as an impacting factor in their study, Coleman and Slingsby (83) speculated that experience, and thus increased awareness, could be a reason for greater pain evaluation for certain conditions between credentialled vs. student nurses. Finally, more women are entering veterinary professions making it difficult to separate the effect of age from gender when evaluating pain perception.

As noted, in the current study the perception of pain influenced the likelihood of providing pain mitigation for almost all procedures and conditions. It is interesting to consider if the perception of pain aligns with the actual alleviation of pain for a variety of common procedures and conditions. Huxley and Whay (17) demonstrated that pain scores for selected procedures in both adult cattle and calves were significantly greater in survey respondents that did provide analgesic drugs compared with respondents that did not. Remnant et al. (25) reported that generally, clinicians more commonly used non-steroidal anti-inflammatory drugs (NSAIDs) for conditions that were rated as more painful, although they did find that for some procedures despite having similar pain scores to other conditions NSAIDs were not as commonly used (e.g., surgical castration and disbudding in calves). Johnstone et al. (19), reporting on the same population as the current study, showed that the provision of pain mitigation for surgical castration varied across age groups; 21.7% of respondents “always” or “most of the time” provided analgesic with surgical castration for calves under 2 months of age compared with 60.3% of respondents for cattle greater than 12 months of age. In a survey study of Canadian veterinarians, Hewson et al., (18) expressed the provision of analgesia as a percentage of calves receiving analgesia and showed that 6.9% and 18.7% of beef and dairy calves six months of age or less received analgesia and 19.9% and 33.2% of beef and dairy calves greater than six months of age received analgesia for castration. Tshoner et al., (89) reported a greater frequency of use of both local and systemic analgesia by veterinarians as compared with producers for surgical castration in calves but also reported that surgical castration was rated as one of the most painful procedures out of the those included in the survey; although it is challenging to compare across studies due to differences in methodology, in the current study, the pain associated with surgical castration was not rated as high. Despite substantial evidence that surgical castration causes pain in cattle (e.g., 11–13, 54, 57, 61–63) and some regulatory guidance existing in countries outside of the United States, the provision of pain mitigation for surgical castration, although increasing has not reached full adoption as a consistently implemented best management practice.

Dehorning and disbudding are two additional procedures that have garnered significant research attention in regard to pain mitigation (14–16). Similar to trends seen with castration, Hewson et al. (18), found that veterinarians who perceived dehorning to be painful were more likely to use analgesia. Related, Wikman et al. (40) reported that dairy producers who perceived disbudding to be more painful, were more attentive to alleviating pain associated with the procedure and thus generally more considerate of cattle pain. These results across studies highlight the importance of the relationship between pain perception and provision of analgesia. One difference worth identifying is that some of these studies demonstrate a greater use of pain mitigation for dehorning or disbudding as compared with those reported for castration. Saraceni et al. (33) reported that 43% of Wisconsin producers provided pain control medication to calves for disbudding; the study was conducted prior to the disbudding requirements in FARM 4.0 (31). Hewson et al. (18) reported a greater percentage of calves receiving pain mitigation (76% dairy, 54% beef) for dehorning than for castration. Winder et al., (21) reported greater use of pain mitigation (i.e., local anesthetic, sedation or NSAIDs) for disbudding in veterinarians as compared with producers in a Canadian survey. Future research should consider exploring techniques used to assess pain as perhaps differences in identifying (or not identifying pain) are impacting the perception of how painful something is. Pain perception could also be impacted by the observable reaction of the animal to the procedure and the subsequent observer interpretation, (e.g., observing vocalizations as compared to foot stomps or head flicks).

Branding is another painful procedure (90, 91) performed on cattle to permanently identify individuals using thermal injury (e.g., hot iron or freeze) that causes tissue damage and is relatively less studied as compared with castration and dehorning in the context of pain mitigation. Branding is a practice that is required in certain regions of the United States to demonstrate ownership, aid in identification when animals are in mixed groups, and protect against theft and there are limited permanent alternatives. In the current study, “severe pain” was the most common pain category selected for hot iron branding. In a survey study of Canadian producers by Moggy et al., (22), the majority of respondents agreed that branding was painful if no pain mitigation was administered but interestingly, in the Moggy et al., (22) study only 4% (2) of respondents provided pain management during branding. Several more recent studies have begun to explore the effectiveness of pain mitigation on pain response associated with branding in cattle (55, 92, 93). The logistics of implementing pain management (e.g., pasture environment, handling facilities, available labor) on a cow-calf operations where branding would most commonly occur can be challenging which may be a reason for reduced pain management implementation rates. Additionally, some studies have explored freeze branding as a less painful alternative to hot iron branding (59, 94–96), and although research has indicated reduced vocalizations, tail flicks, kicks, falls and cortisol response in freeze branded cattle (59, 96), there are limitations to the technique, such as increased time needed to perform the procedure and ineffectiveness of the brand on light colored animals (97), that have likely limited the widespread adoption of the practice. In the current study, “mild pain” was the most commonly selected pain category associated with freeze branding. This sentiment aligns with the current scientific knowledge indicating freeze branding as a less painful alternative to hot iron branding.

Alleviating pain associated with disease states in cattle is also a critical and current area of research. Considerable work has examined the pain associated with lameness in cattle, particularly in the dairy industry (9, 10, 98). In the current study approximately three-quarters of respondents identified the pain associated with lameness as moderate or severe across age categories. The majority of dairy farmers surveyed in a study in the United Kingdom considered “pain and suffering for the cow” a very important or extremely important consequence of lameness (99). Across studies, including the present study, there is a relationship between the likelihood of providing treatment for lameness and the perceived level of pain. Similar to the present study, a survey of Swiss dairy farmers, claw trimmers, and cattle veterinarians found that respondents who reported greater pain scores for sole ulcer treatment more often considered providing local anesthesia as compared with those that reported lower associated pain (100). In-depth interviews of dairy farmers revealed that the speed of treatment for lameness was influenced by the perceived severity of lameness, although “treatment” was not defined and could include a variety of strategies, one being analgesia (101). Tunstall et al., (102) conducted a study with beef producers in the United Kingdom and through thematic analysis demonstrated that some producers indicated they would not provide pain relief to beef cattle, even when they perceived lameness to be painful. The efficacy of systemic analgesia is not as clear; while anti-inflammatories have been effective for pain relief in induced-lameness models, they have varying levels of efficacy in field trials and have been associated with only mild relief of discomfort (reviewed by 10). Future research and outreach efforts should focus on identifying signs of pain that accompany lameness to improve identification of lame animals, demonstrating that lameness is painful to encourage producers and veterinarians to provide pain relief, and investigating effective analgesics for lame cattle to ensure cattle receive appropriate treatment.

Bovine respiratory disease is a common disease in both the dairy and beef cattle industries (44–46). Despite its pervasiveness, there has been limited research exploring pain associated with BRD in cattle (60). In the current study, nearly one-third of respondents indicated that BRD caused mild or no pain in cattle across age categories. Although available data is sparse, recent work has begun to explore the pain associated with BRD in cattle. Martin et al. (60) demonstrated that calves challenged with *Mannheimia haemolytica* and treated with an NSAID (transdermal flunixin) had increased activity levels and decreased pain scores measured on a visual analog scale compared to challenged calves that received a placebo. Furthermore, substance *P*, which is a neuropeptide that is increased when the nervous system responds to painful stimuli, was greater in calves with pneumonia due to inoculation with *Mannheimia haemolytica* compared to control calves (103). Indeed, evidence thus far in cattle (60, 103), combined with human literature (104, 105) indicates that respiratory disease is a painful condition. Although there is growing evidence that BRD is painful, a recent survey of veterinarians that are involved with treatment decisions for preweaned calves found that 44% of veterinarians reported that NSAIDs were the most important ancillary treatment for BRD (106), indicating that many veterinarians are not administering NSAIDs to calves with BRD. The present study demonstrated that the likelihood of providing pain relief was associated with the level of perceived pain. Further investigation of pain associated with BRD would be useful to help increase awareness among veterinarians and producers that BRD is painful, thus encouraging the use of pain mitigation when treating BRD.

Although the sample size in this survey was substantial, the authors would like to address areas of potential sampling bias. The survey was delivered electronically and therefore this may have limited the breadth of respondents reached with the survey; individuals without access to the internet or that were not part of membership organization listservs would have been underrepresented in the study population. Future work could examine using multi-medium survey distribution to reach a wider group of veterinarians and producers. Additionally, individuals who responded to the survey may have had a significant interest in pain mitigation, either in support of or questioning of, and thus this could have introduced sampling bias.

Addressing animal welfare concerns through ensuring adequate pain management is important in the livestock industry. The use of pain mitigation is also important to consumers; in a national web-based survey conducted in the United States, over 80% of consumers found it very or somewhat important that pain relief be provided for castration and removal of horns or horn buds (107). The link between perception of pain and the use of local and systemic analgesia requires robust assessment of pain in livestock, irrespective of age of the animal. Further development of pain measurement is needed to assess the level of pain experienced in younger animals to ensure pain management is appropriate. Better understanding of the differences between producer and veterinary assessment of pain in livestock is needed to bridge gaps in pain mitigation between the groups. Further, it may be useful to delve into the gender differences regarding pain perception to understand how to provide appropriate training to producers and veterinarians engaged in pain management in livestock. Without clear and consistent measures to assess pain in livestock, it is not possible to know if pain management approaches are working and to properly link perceptions of pain by humans is addressing that experienced by the animals.

## Data Availability

The raw data supporting the conclusions of this article will be made available by the authors, without undue reservation.
