# Supplementary material for: A nationwide survey on producer and veterinarian perceptions of the painfulness of procedures and disease states in dairy and beef cattle
Source: Front Pain Res (Lausanne). 2023 Feb 1;4:1059224. doi: 10.3389/fpain.2023.1059224 (PMC9929155; doi:10.3389/fpain.2023.1059224)
Supplement: Supplementary file 1 [file Table1.pdf]

Supplementary Table 1. The p-values for the likelihood ratio tests corresponding to the results in Table 3.

Procedure or Condition	Cattle age (mo)	Pain perception of procedure	Gender	Role in cattle industry
Abdominal surgery	< 2	<0.00001	0.12	<0.00001
	2 to 12	<0.00001	0.018	<0.00001
	> 12	0.00017	0.0011	<0.00001
Surgical castration	< 2	<0.00001	0.0020	0.0029
	2 to 12	<0.00001	0.00028	0.00001
	> 12	<0.00001	0.00011	<0.00001
Band castration	< 2	<0.00001	0.12	0.0018
	2 to 12	<0.00001	0.045	0.193
	> 12	-	-	-
Hot iron dehorning	< 2	<0.00001	<0.00001	<0.00001
	2 to 12	<0.00001	<0.00001	<0.00001
	> 12	<0.00001	0.00002	<0.00001
Paste disbudding	< 2	<0.00001	<0.00001	<0.00001
	2 to 12	<0.00001	0.00054	<0.00001
	> 12	-	-	-

Supplementary Table 2. The p-values for the likelihood ratio tests corresponding to the results in Table 4.

Procedure	Cattle age (mo)	Pain perception of procedure	Gender	Role in cattle industry
surgery	< 2	<0.00001	0.0022	0.096
	2 to 12	<0.00001	0.0021	0.0018
	> 12	<0.00001	0.23	0.17
Surgical castration	< 2	<0.00001	0.017	<0.00001
	2 to 12	<0.00001	0.0030	<0.00001
	> 12	<0.00001	0.0006	<0.00001
Band castration	< 2	<0.00001	0.0018	0.00001
	2 to 12	<0.00001	0.0023	0.0004
	> 12	-	-	-
Hot iron dehorning	< 2	<0.00001	0.00009	<0.00001
	2 to 12	<0.00001	0.013	<0.00001
	> 12	<0.00001	0.013	<0.00001
Paste disbudding	< 2	<0.00001	0.00008	<0.00001
	2 to 12	<0.00001	0.034	<0.00001
	> 12	-	-	-
Hot iron branding	< 2	<0.00001	0.28	0.053
	2 to 12	<0.00001	0.33	0.37
	> 12	0.30	0.035	0.36
Freeze branding	< 2	<0.00001	0.85	0.005
	2 to 12	<0.00001	0.91	0.087
	> 12	0.049	0.11	0.30
Bovine respiratory disease	< 2	<0.00001	<0.00001	<0.00001
	2 to 12	<0.00001	<0.00001	<0.00001
	> 12	0.011	0.23	0.39
Lameness	< 2	<0.00001	0.00043	<0.00001
	2 to 12	<0.00001	<0.00001	<0.00001
	> 12	0.17	0.12	0.40
