# Supplementary material for: A nationwide survey on producer and veterinarian perceptions of the painfulness of procedures and disease states in dairy and beef cattle
Source: Front Pain Res (Lausanne). 2023 Feb 1;4:1059224. doi: 10.3389/fpain.2023.1059224 (PMC9929155; doi:10.3389/fpain.2023.1059224)
Supplement: Supplementary file 2 [file Datasheet1.pdf]

Cattle Pain Management Survey

Q1 Where is your operation located?

❍ Alabama	❍ Louisiana	❍ Oklahoma
❍ Alaska	❍ Maine	❍ Oregon
❍ Arizona	❍ Maryland	❍ Pennsylvania
❍ Arkansas	❍ Massachusetts	❍ Puerto Rico
❍ California	❍ Michigan	❍ Rhode Island
❍ Colorado	❍ Minnesota	❍ South Carolina
❍ Connecticut	❍ Mississippi	❍ South Dakota
❍ Delaware	❍ Missouri	❍ Tennessee
❍ District of Columbia	❍ Montana	❍ Texas
❍ Florida	❍ Nebraska	❍ Utah
❍ Georgia	❍ Nevada	❍ Vermont
❍ Hawaii	❍ New Hampshire	❍ Virginia
❍ Idaho	❍ New Jersey	❍ Washington
❍ Illinois	❍ New Mexico	❍ West Virginia
❍ Indiana	❍ New York	❍ Wisconsin
❍ Iowa	❍ North Carolina	❍ Wyoming
❍ Kansas	❍ North Dakota	❍ I do not reside in the United States
❍ Kentucky	❍ Ohio	

Q2 Are you?

❍ Male
❍ Female

Q3 Which of the following best describes your involvement with the cattle industry? (Select all that apply.)

❑ Producer (beef or dairy)
❑ Veterinarian
❑ Other (please specify):____

Display This Question: If Q3 = Producer (beef or dairy)

Q4 Which of following apply to your operation? (Select all that apply.)

❑ Dairy
❑ Calf Ranch
❑ Feedlot
❑ Stocker/Backgrounder
❑ Cow-calf
❑ Other (please specify):____

Cattle Pain Management Survey

Display This Question: If Q3 = Veterinarian

Q5 Which of the following cattle operation types does your practice serve? (Select all that apply.)

- ❑ Dairy
- ❑ Calf Ranch
- ❑ Feedlot
- ❑ Stocker/Backgrounder
- ❑ Cow-calf
- ❑ Other (please specify):____

Display This Question: If Q3 = Producer (beef or dairy)

Q6 How many head of the following do you have in inventory?

Beef cows	Less than 50 cows	50 to 199 cows	200 to 499 cows	500 to 999 cows	1,000 cows or more				
Cattle on feed	Less than 1,000 head	1,000 to 4,999 head	5,000 to 9,999 head	10,000 to 29,999 head	30,000 to 49,999 head	50,000 to 69,999 head	70,000 head or more		
Stockers/Backgrounders	Less than 500 head	500 to 999 head	1,000 to 1,999 head	2,000 to 4,999 head	5,000 head or more				
Dairy calves	Less than 5,000 head	5,000 to 9,999 head	10,000 to 19,999 head	20,000 head or more					
Dairy cows	Less than 50 cows	50 to 99 cows	100 t0 199 cows	200 to 499 cows	500 to 999 cows	1,000 to 1,999 cows	2,000 to 4,999 cows	5,000 to 9,999 cows	10,000 cows or more

Q7 How old are you?

- ❍ Under 20
- ❍ 21 to 30
- ❍ 31 to 40
- ❍ 41 to 50
- ❍ 51 to 60
- ❍ 61 to 70
- ❍ Over 70 years old

Cattle Pain Management Survey

Display This Question: If Q3 = Producer (beef or dairy)

Q8 Which of the following best describes your relationship with your cattle operation?

- ❍ Owner
- ❍ Manager
- ❍ Foreman
- ❍ Supervisor
- ❍ Herdsman
- ❍ Farm/Ranch Hand
- ❍ Milker
- ❍ Other (please specify):____

Display This Question: If Q3 = Producer (beef or dairy)

Q9 What is the highest degree or level of school you have completed? If currently enrolled, highest degree received.

- ❍ Did not complete high school
- ❍ High School
- ❍ Some College, no degree
- ❍ Trade/technical/vocational training
- ❍ Associate Degree
- ❍ Bachelor's Degree
- ❍ Master's Degree
- ❍ Professional Degree (JD, MD)
- ❍ Doctorate Degree (PhD)

Display This Question: If Q3 = Veterinarian

Q10 When did you graduate from veterinary school?

- ❍ Prior to 1970
- ❍ 1970-1980
- ❍ 1981-1990
- ❍ 1991-2000
- ❍ 2001-2010
- ❍ 2011-2018

Display This Question: If Q3 = Producer (beef or dairy)

Q11 How is your cattle operation classified?

- ❍ Conventional operation
- ❍ USDA Certified Organic
- ❍ Naturally-raised (non-hormone, antibiotic free)
- ❍ Other (please specify):____

Cattle Pain Management Survey

Display This Question: If Q3 = Producer (beef or dairy)

Q12 Are any of your cattle part of a verified program (such as Certified Humane, Source and Age Verified, American Grassfed, Process Verified)?

- ❍ Yes
- ❍ No

Display This Question: If Q12 = Yes

Q13 Which program or programs are your cattle part of? ________________________________

Q14 In my operation or practice, I currently use pain management (local anesthetic (lidocaine), systemic analgesia (e.g. Aspirin/ Banamine®) with: (Select all that apply.)

- ❑ Calves less than 2 months of age
- ❑ Calves 2 to 12 months of age
- ❑ Adult cattle (more than 12 months of age)
- ❑ Do not use pain management on any of my cattle

Q15 How likely are you to use a local anesthetic (lidocaine) for the following conditions in calves less than 2 months of age?

	Never	Sometimes	About half the time	Most of the time	Always	Would not perform this procedure
Surgical castration	❍	❍	❍	❍	❍	❍
Band castration	❍	❍	❍	❍	❍	❍
Dehorning	❍	❍	❍	❍	❍	❍
Abdominal surgery	❍	❍	❍	❍	❍	❍

Q16 How likely are you to use a local anesthetic (lidocaine) for the following conditions in calves 2 to 12 months of age?

	Never	Sometimes	About half the time	Most of the time	Always	Would not perform this procedure
Surgical castration	❍	❍	❍	❍	❍	❍
Band castration	❍	❍	❍	❍	❍	❍
Dehorning	❍	❍	❍	❍	❍	❍
Abdominal surgery	❍	❍	❍	❍	❍	❍

Q17 How likely are you to use a local anesthetic (lidocaine) for the following conditions in ADULT cattle (over 12 months of age)?

	Never	Sometimes	About half the time	Most of the time	Always	Would not perform this procedure
Surgical castration	❍	❍	❍	❍	❍	❍
Dehorning	❍	❍	❍	❍	❍	❍
Abdominal surgery (including DA and Cesarean)	❍	❍	❍	❍	❍	❍

Cattle Pain Management Survey

Q18 How likely are you to use a systemic pain relief drug (analgesic) for the following conditions in calves less than 2 months of age?

	Never	Sometimes	About half the time	Most of the time	Always	Would not perform this procedure
Surgical castration	❍	❍	❍	❍	❍	❍
Band castration	❍	❍	❍	❍	❍	❍
Dehorning	❍	❍	❍	❍	❍	❍
Abdominal surgery	❍	❍	❍	❍	❍	❍
Branding	❍	❍	❍	❍	❍	❍
BRD (Pneumonia)	❍	❍	❍	❍	❍	❍
Lameness	❍	❍	❍	❍	❍	❍

Q19 How likely are you to use a systemic pain relief drug (analgesic) for the following conditions in calves 2 to 12 months of age?

	Never	Sometimes	About half the time	Most of the time	Always	Would not perform this procedure
Surgical castration	❍	❍	❍	❍	❍	❍
Band castration	❍	❍	❍	❍	❍	❍
Dehorning	❍	❍	❍	❍	❍	❍
Abdominal surgery	❍	❍	❍	❍	❍	❍
Branding	❍	❍	❍	❍	❍	❍
BRD (Pneumonia)	❍	❍	❍	❍	❍	❍
Lameness	❍	❍	❍	❍	❍	❍

Q20 How likely are you to use a systemic pain relief drug (analgesic) for the following conditions in ADULT cattle?

	Never	Sometimes	About half the time	Most of the time	Always	Would not perform this procedure
Surgical castration	❍	❍	❍	❍	❍	❍
Dehorning	❍	❍	❍	❍	❍	❍
Abdominal surgery (including DA and Cesarean)	❍	❍	❍	❍	❍	❍
Branding	❍	❍	❍	❍	❍	❍
BRD (Pneumonia)	❍	❍	❍	❍	❍	❍
Lameness	❍	❍	❍	❍	❍	❍
Mastitis	❍	❍	❍	❍	❍	❍

Cattle Pain Management Survey

Q21 Which pain relief drugs (analgesics) do you have knowledge of and feel comfortable using in your operation or practice? (Select all that apply.)

- ❑ Lidocaine
- ❑ Oral Meloxicam
- ❑ Meloxicam Injection (Metacam® Injection)
- ❑ Flunixin (e.g. Banamine®) Injection
- ❑ Flunixin (e.g. Banamine®) pour-on
- ❑ Aspirin
- ❑ Phenylbutazone
- ❑ Ketoprofen (Anafen® Injection)
- ❑ Other (please specify):____
- ❑ None of these

Q22 How painful would you consider the following conditions to be in calves less than 2 months of age?

	No pain	Mild	Moderate	Severe	Very severe	Worst pain imaginable
Surgical castration	❍	❍	❍	❍	❍	❍
Band castration	❍	❍	❍	❍	❍	❍
Hot iron dehorning/ disbudding	❍	❍	❍	❍	❍	❍
Paste dehorning/ disbudding	❍	❍	❍	❍	❍	❍
Abdominal surgery/ umbilical abscess	❍	❍	❍	❍	❍	❍
Freeze branding	❍	❍	❍	❍	❍	❍
Hot iron branding	❍	❍	❍	❍	❍	❍
BRD (Pneumonia)	❍	❍	❍	❍	❍	❍
Lameness	❍	❍	❍	❍	❍	❍
Ear tagging	❍	❍	❍	❍	❍	❍
Skin lesions/Dermatitis	❍	❍	❍	❍	❍	❍
Limb fracture	❍	❍	❍	❍	❍	❍
Calving without assistance	❍	❍	❍	❍	❍	❍
Calving with assistance	❍	❍	❍	❍	❍	❍
Diarrhea (scours)	❍	❍	❍	❍	❍	❍

Cattle Pain Management Survey

Q23 How painful would you consider the following conditions to be in calves 2 to 12 months of age?

	No pain	Mild	Moderate	Severe	Very severe	Worst pain imaginable
Surgical castration	❍	❍	❍	❍	❍	❍
Band castration	❍	❍	❍	❍	❍	❍
Hot iron dehorning/ disbudding	❍	❍	❍	❍	❍	❍
Paste dehorning/ disbudding	❍	❍	❍	❍	❍	❍
Abdominal surgery/ umbilical abscess	❍	❍	❍	❍	❍	❍
Freeze branding	❍	❍	❍	❍	❍	❍
Hot iron branding	❍	❍	❍	❍	❍	❍
BRD (Pneumonia)	❍	❍	❍	❍	❍	❍
Lameness	❍	❍	❍	❍	❍	❍
Ear tagging	❍	❍	❍	❍	❍	❍
Skin lesions/Dermatitis	❍	❍	❍	❍	❍	❍
Limb fracture	❍	❍	❍	❍	❍	❍
Diarrhea	❍	❍	❍	❍	❍	❍

Q24 How painful would you consider the following conditions to be in ADULT cattle (older than 12 months of age)?

	No pain	Mild	Moderate	Severe	Very severe	Worst pain imaginable
Surgical castration	❍	❍	❍	❍	❍	❍
Band castration	❍	❍	❍	❍	❍	❍
Hot iron dehorning/ disbudding	❍	❍	❍	❍	❍	❍
Abdominal surgery (including DA and Cesarean)	❍	❍	❍	❍	❍	❍
Freeze branding	❍	❍	❍	❍	❍	❍
Hot iron branding	❍	❍	❍	❍	❍	❍
BRD (Pneumonia)	❍	❍	❍	❍	❍	❍
Lameness	❍	❍	❍	❍	❍	❍
Ear tagging	❍	❍	❍	❍	❍	❍
Acute Metritis	❍	❍	❍	❍	❍	❍
Acute Mastitis (with fever)	❍	❍	❍	❍	❍	❍
Skin lesions/Dermatitis	❍	❍	❍	❍	❍	❍
Clinical mastitis (no fever)	❍	❍	❍	❍	❍	❍
Calving without assistance	❍	❍	❍	❍	❍	❍
Calving with assistance	❍	❍	❍	❍	❍	❍
Diarrhea	❍	❍	❍	❍	❍	❍

Cattle Pain Management Survey

Q25 How important are the following factors in impacting your decision to use an analgesic drug in adult cattle and calves?

	Not at all important	Slightly important	Moderately important	Very important	Extremely important
FDA Approval status	❍	❍	❍	❍	❍
Cost of the drug	❍	❍	❍	❍	❍
Recommendation of veterinarian (Producers only)	❍	❍	❍	❍	❍
Lack of sedative effect	❍	❍	❍	❍	❍
Duration of Pain Control/ Analgesic effect of drug	❍	❍	❍	❍	❍
Ease of administration	❍	❍	❍	❍	❍
Short Withhold Period	❍	❍	❍	❍	❍
Animal's ability to feel pain	❍	❍	❍	❍	❍
Improving Safety of the caregiver/ operator	❍	❍	❍	❍	❍
Improved production outcomes	❍	❍	❍	❍	❍
How painful I consider the procedure to be	❍	❍	❍	❍	❍
Time of onset of drug activity	❍	❍	❍	❍	❍
Request of producer (Veterinarians only)	❍	❍	❍	❍	❍

Q26 What would you consider an acceptable cost/head for a course of analgesia for the following conditions/procedures for calves less than 2 months of age in your operation or practice? (Select one response for each)

	$0	Less than $5	$5 to $9.99	$10 to $19.99	$20 to 29.99	$30 or more
Surgical castration	❍	❍	❍	❍	❍	❍
Band castration	❍	❍	❍	❍	❍	❍
Abdominal surgery/umbilical hernia repair	❍	❍	❍	❍	❍	❍
Hot iron dehorning/disbudding	❍	❍	❍	❍	❍	❍
Paste dehorning/disbudding	❍	❍	❍	❍	❍	❍
BRD (Pneumonia)	❍	❍	❍	❍	❍	❍
Lameness	❍	❍	❍	❍	❍	❍
Mastitis	❍	❍	❍	❍	❍	❍
Dystocia/Difficult birth	❍	❍	❍	❍	❍	❍

Cattle Pain Management Survey

Q27 What would you consider an acceptable cost/head for a course of analgesia for the following conditions/procedures calves 2 to 12 months of age in your operation or practice? (Select one response for each)

	$0	Less than $5	$5 to $9.99	$10 to $19.99	$20 to 29.99	$30 or more
Surgical castration	❍	❍	❍	❍	❍	❍
Band castration	❍	❍	❍	❍	❍	❍
Abdominal surgery/umbilical hernia repair	❍	❍	❍	❍	❍	❍
Hot iron dehorning/disbudding	❍	❍	❍	❍	❍	❍
Paste dehorning/disbudding	❍	❍	❍	❍	❍	❍
BRD (Pneumonia)	❍	❍	❍	❍	❍	❍
Lameness	❍	❍	❍	❍	❍	❍
Mastitis	❍	❍	❍	❍	❍	❍
Dystocia/Difficult birth	❍	❍	❍	❍	❍	❍
Acute Metritis	❍	❍	❍	❍	❍	❍
Mastitis	❍	❍	❍	❍	❍	❍

Q28 What would you consider an acceptable cost/head for a course of analgesia for the following conditions/procedures ADULT cattle (older than 12 months of age) in your operation or practice? (Select one response for each)

	$0	Less than $5	$5 to $9.99	$10 to $19.99	$20 to 29.99	$30 or more
Surgical castration	❍	❍	❍	❍	❍	❍
Band castration	❍	❍	❍	❍	❍	❍
Abdominal surgery/umbilical hernia repair	❍	❍	❍	❍	❍	❍
Hot iron dehorning/disbudding	❍	❍	❍	❍	❍	❍
Paste dehorning/disbudding	❍	❍	❍	❍	❍	❍
BRD (Pneumonia)	❍	❍	❍	❍	❍	❍
Lameness	❍	❍	❍	❍	❍	❍
Mastitis	❍	❍	❍	❍	❍	❍
Dystocia/Difficult birth	❍	❍	❍	❍	❍	❍
Acute Metritis	❍	❍	❍	❍	❍	❍
Mastitis	❍	❍	❍	❍	❍	❍

Cattle Pain Management Survey

Q29 Select the response that best reflects your opinion:

	Agree	Not sure	Disagree
Analgesics may mask deterioration in the animal's condition	❍	❍	❍
Cattle benefit from receiving analgesic drugs as part of their treatment	❍	❍	❍
Cattle that are experiencing a fever are in pain	❍	❍	❍
Some pain is necessary to stop the animal becoming too active	❍	❍	❍
Cattle recover faster if given analgesic drugs	❍	❍	❍
Drug side effects limit the usefulness of giving analgesics to cattle	❍	❍	❍
Most farmers are willing to pay the costs involved with giving analgesics to cattle.	❍	❍	❍
The benefits of the analgesia outweighs the cost of the analgesia	❍	❍	❍
Farmers would like cattle to receive analgesia but cost is a major issue	❍	❍	❍
U.S/USDA/FDA regulations limit my ability to use analgesic drugs in cattle	❍	❍	❍

Q30 How has your use of analgesics changed in the last 10 years?

- ❍ Increased use
- ❍ Stayed the same
- ❍ Decreased use

Display This Question: If Q30 = Increased use

Q31 Why has your use of analgesics increased? (Select all that apply.)

- ❑ New evidence of analgesic effectiveness
- ❑ Requirement of a quality assurance program
- ❑ Decreased prices for analgesics
- ❑ Change in your perception of pain in cattle
- ❑ Changing farmer or veterinarian attitudes
- ❑ Change in practice or operation protocols
- ❑ Influence from colleagues/fellow producers
- ❑ Mandated by a retailer or packer
- ❑ Maintain consumer confidence in livestock production practices
- ❑ Cattle that receive analgesia look better than cattle that don't
- ❑ Cattle that receive analgesia have improved health and performance

Cattle Pain Management Survey

Display This Question: If Q30 = Decreased use

Q32 Why has your use of analgesics decreased? (Select all that apply.)

- ❑ Currently available analgesic drugs are not effective at reducing pain
- ❑ Currently available analgesic drugs are inconvenient to administer
- ❑ Currently available analgesic drugs do not last long enough after 1 dose to justify their use
- ❑ Currently available analgesic drugs are too expensive
- ❑ I do not know the meat and milk withhold periods for the analgesic drugs
- ❑ Currently available drugs do not improve health and performance
- ❑ I am not comfortable using an analgesic unless it has been approved by FDA

Display This Question: If Q3 = Producer (beef or dairy)

Q33 If you and your attending veterinarian disagree about the use or lack of use of pain management for your cattle, how likely would you proceed with the following courses of action? (Please select a choice for each course of action.)

	Extremely unlikely	Somewhat unlikely	Neither likely nor unlikely	Somewhat likely	Extremely likely
Find a different veterinarian who agrees with you	❍	❍	❍	❍	❍
Take a chance and try what the veterinarian suggests	❍	❍	❍	❍	❍
Argue with veterinarian until they do what you ask	❍	❍	❍	❍	❍
Do what you want without the veterinarian knowing	❍	❍	❍	❍	❍
Ask to be provided more information about pain in cattle/ perform your own research to either support or change your opinion	❍	❍	❍	❍	❍

Cattle Pain Management Survey

Display This Question: If Q3 = Veterinarian

Q34 If you and your client disagree about the use or lack of use of pain management for their cattle, how likely would you proceed with the following courses of action? (Please select a choice for each course of action.)

	Extremely unlikely	Somewhat unlikely	Neither likely nor unlikely	Somewhat likely	Extremely likely
Terminate the VCPR/ relationship with client	❍	❍	❍	❍	❍
Do what the client asks	❍	❍	❍	❍	❍
Argue with the client until they agree with your advice	❍	❍	❍	❍	❍
Do what you want and charge the client accordingly	❍	❍	❍	❍	❍
Perform your own research to either support or change your opinion or try to understand the client's wishes.	❍	❍	❍	❍	❍

Display This Question: If Q3 = Veterinarian

Q35 How often do disagreements about the use of pain management in cattle affect your relationship with your producer?

- ❍ Daily
- ❍ Once weekly
- ❍ Few times monthly
- ❍ Several times a year
- ❍ Less than once a year
- ❍ Never

Display This Question: If Q3 = Producer (beef or dairy)

Q36 How often do disagreements about the use of pain management in cattle affect your relationship with your veterinarian?

- ❍ Daily
- ❍ Once weekly
- ❍ Few times monthly
- ❍ Several times a year
- ❍ Less than once a year
- ❍ Never

Q37 Do you consider that your knowledge about recognizing and treating pain in adult cattle and calves is adequate?

- ❍ Yes
- ❍ No

Q38 Where do you feel you have obtained most of your knowledge about recognizing and treating pain in adult cattle and calves?

- ❍ FFA/ 4-H training
- ❍ College classes
- ❍ Journals / articles
- ❍ Continuing education
- ❍ Personal Experience
- ❍ Online training modules
- ❍ Commercial literature / data sheets
- ❍ Other (please specify):____

Display This Question: If Q3 = Veterinarian

Q39 Does the type of illness or procedure affect the likelihood of using a specific analgesic drug in the cattle you treat?

- ❍ Yes
- ❍ No

Display This Question: If Q39 = No

Q40 How likely are you to consider using the following analgesic drugs in cattle?

	Extremely unlikely	Somewhat unlikely	Neither likely nor unlikely	Somewhat likely	Extremely likely
Lidocaine	❍	❍	❍	❍	❍
Oral Meloxicam	❍	❍	❍	❍	❍
Meloxicam Injection	❍	❍	❍	❍	❍
Flunixin (Banamine) Injection	❍	❍	❍	❍	❍
Flunixin (Banamine) pour-on	❍	❍	❍	❍	❍
Aspirin	❍	❍	❍	❍	❍
Phenylbutazone	❍	❍	❍	❍	❍
Ketoprofen	❍	❍	❍	❍	❍
Other (please specify):	❍	❍	❍	❍	❍

Cattle Pain Management Survey

Display Q41 to Q46: If Q39 = Yes

Q41 How likely are you to consider using the following analgesic drugs in calves at the time of dehorning?

	Extremely unlikely	Somewhat unlikely	Neither likely nor unlikely	Somewhat likely	Extremely likely
Lidocaine	❍	❍	❍	❍	❍
Oral Meloxicam	❍	❍	❍	❍	❍
Meloxicam Injection	❍	❍	❍	❍	❍
Flunixin (Banamine) Injection	❍	❍	❍	❍	❍
Flunixin (Banamine) pour-on	❍	❍	❍	❍	❍
Aspirin	❍	❍	❍	❍	❍
Phenylbutazone	❍	❍	❍	❍	❍
Ketoprofen	❍	❍	❍	❍	❍
Other (please specify):	❍	❍	❍	❍	❍

Q42 How likely are you to consider using the following analgesic drugs in calves at the time of castration?

	Extremely unlikely	Somewhat unlikely	Neither likely nor unlikely	Somewhat likely	Extremely likely
Lidocaine	❍	❍	❍	❍	❍
Oral Meloxicam	❍	❍	❍	❍	❍
Meloxicam Injection	❍	❍	❍	❍	❍
Flunixin (Banamine) Injection	❍	❍	❍	❍	❍
Flunixin (Banamine) pour-on	❍	❍	❍	❍	❍
Aspirin	❍	❍	❍	❍	❍
Phenylbutazone	❍	❍	❍	❍	❍
Ketoprofen	❍	❍	❍	❍	❍
Other (please specify):	❍	❍	❍	❍	❍

Q43 How likely are you to consider using the following analgesic drugs in calves at the time of branding?

	Extremely unlikely	Somewhat unlikely	Neither likely nor unlikely	Somewhat likely	Extremely likely
Lidocaine	❍	❍	❍	❍	❍
Oral Meloxicam	❍	❍	❍	❍	❍
Meloxicam Injection	❍	❍	❍	❍	❍
Flunixin (Banamine) Injection	❍	❍	❍	❍	❍
Flunixin (Banamine) pour-on	❍	❍	❍	❍	❍
Aspirin	❍	❍	❍	❍	❍
Phenylbutazone	❍	❍	❍	❍	❍
Ketoprofen	❍	❍	❍	❍	❍
Other (please specify):	❍	❍	❍	❍	❍

Cattle Pain Management Survey

Q44 How likely are you to consider using the following analgesic drugs in lame cattle?

	Extremely unlikely	Somewhat unlikely	Neither likely nor unlikely	Somewhat likely	Extremely likely
Lidocaine	❍	❍	❍	❍	❍
Oral Meloxicam	❍	❍	❍	❍	❍
Meloxicam Injection	❍	❍	❍	❍	❍
Flunixin (Banamine) Injection	❍	❍	❍	❍	❍
Flunixin (Banamine) pour-on	❍	❍	❍	❍	❍
Aspirin	❍	❍	❍	❍	❍
Phenylbutazone	❍	❍	❍	❍	❍
Ketoprofen	❍	❍	❍	❍	❍
Other (please specify):	❍	❍	❍	❍	❍

Q45 How likely are you to consider using the following analgesic drugs in mastitis cattle?

	Extremely unlikely	Somewhat unlikely	Neither likely nor unlikely	Somewhat likely	Extremely likely
Lidocaine	❍	❍	❍	❍	❍
Oral Meloxicam	❍	❍	❍	❍	❍
Meloxicam Injection	❍	❍	❍	❍	❍
Flunixin (Banamine) Injection	❍	❍	❍	❍	❍
Flunixin (Banamine) pour-on	❍	❍	❍	❍	❍
Aspirin	❍	❍	❍	❍	❍
Phenylbutazone	❍	❍	❍	❍	❍
Ketoprofen	❍	❍	❍	❍	❍
Other (please specify):	❍	❍	❍	❍	❍

Q46 How likely are you to consider using the following analgesic drugs in cows at calving?

	Extremely unlikely	Somewhat unlikely	Neither likely nor unlikely	Somewhat likely	Extremely likely
Lidocaine	❍	❍	❍	❍	❍
Oral Meloxicam	❍	❍	❍	❍	❍
Meloxicam Injection	❍	❍	❍	❍	❍
Flunixin (Banamine) Injection	❍	❍	❍	❍	❍
Flunixin (Banamine) pour-on	❍	❍	❍	❍	❍
Aspirin	❍	❍	❍	❍	❍
Phenylbutazone	❍	❍	❍	❍	❍
Ketoprofen	❍	❍	❍	❍	❍
Other (please specify):	❍	❍	❍	❍	❍
